# Comparison of effectiveness and cost for different HIV screening strategies implemented at large urban medical centre in the United States

**DOI:** 10.1002/jia2.25554

**Published:** 2020-10-29

**Authors:** Britt Skaathun, Mai T Pho, Harold A Pollack, Samuel R Friedman, Moira C McNulty, Eleanor E Friedman, Jessica Schmitt, David Pitrak, John A Schneider

**Affiliations:** ^1^ Department of Infectious Diseases and Global Public Health University of California San Diego CA USA; ^2^ Department of Public Health Sciences University of Chicago Chicago IL USA; ^3^ Chicago Center for HIV Elimination Chicago IL USA; ^4^ Department of Medicine University of Chicago Chicago IL USA; ^5^ School of Social Service Administration University of Chicago Chicago IL USA; ^6^ Department of Population Health New York University Medical School New York NY USA

**Keywords:** HIV, screening, cost, social networks, undiagnosed HIV infection

## Abstract

**Introduction:**

Incident HIV infections persist in the United States (U.S.) among marginalized populations. Targeted and cost‐efficient testing strategies can help in reaching HIV elimination. This analysis compares the effectiveness and cost of three HIV testing strategies in a high HIV burden area in the U.S. in identifying new HIV infections.

**Methods:**

We performed a cost analysis comparing three HIV testing strategies in Chicago: (1) routine screening (RS) in an inpatient and outpatient setting, (2) modified partner services (MPS) among networks of the recently HIV infected and diagnosed, and (3) a respondent drive sampling (RDS)‐based social network (SN) approach targeting young African‐American men who have sex with men. All occurred at the same academic medical centre during the following times: routine testing, 2011 to 2016; MPS, 2013 to 2016; SN: 2013 to 2014. Costs were in 2016 dollars and included personnel, HIV testing, training, materials, overhead. Outcomes included cost per test, HIV‐positive test and new diagnosis. Sensitivity analyses were performed to assess the impact of population demographics.

**Results:**

The RS programme completed 57,308 HIV tests resulting in 360 (0.6%) HIV‐positive tests and 165 new HIV diagnoses (0.28%). The MPS completed 146 HIV tests, resulting in 79 (54%) HIV‐positive tests and eight new HIV diagnoses (5%). The SN strategy completed 508 HIV tests, resulting in 210 (41%) HIV‐positive tests and 37 new HIV diagnoses (7.2%). Labour accounted for the majority of costs in all strategies. The estimated cost per new HIV diagnosis was $16,773 for the RS programme, $61,418 for the MPS programme and $15,683 for the SN testing programme. These costs were reduced for the RS and MPS strategies in sensitivity analyses limiting testing efficacy to the highest prevalence patient populations ($2,841 and $33,233 respectively).

**Conclusions:**

The SN strategy yielded the highest proportion of new diagnoses, followed closely by the MPS programme. Both the SN strategy and RS programme were comparable in the cost per new diagnosis. A simultaneous approach that consists of RS in combination with SN testing may be most effective for identifying new HIV infections in settings with heterogeneous epidemics with both high rates of HIV prevalence and HIV testing.

## 1 INTRODUCTION

The United States (U.S.) continues to experience new HIV infections; the number of new HIV diagnoses remains stable at around 40,000 cases per year [1]. U.S. Health and Human Services recently established eliminating new HIV infections by 2030 as a goal [2]. Rapid diagnosis and treatment of individuals infected with HIV are two components identified for reaching this goal. However, specifics related to increasing HIV testing and treatment of new positives has not been provided.

In 2011, the Centers for Disease Control and Prevention (CDC) established a “High‐Impact HIV Prevention” approach to reducing HIV infection in response to limited HIV prevention resources [3]. This approach considers several factors including effectiveness, cost and feasibility of full‐scale implementation [3], and is increasingly relevant as federal HIV prevention resources remain constrained, despite the “Ending the HIV Epidemic” initiative [4, 5].

Limited data exist concerning real‐world costs of implementing varied HIV screening strategies. Concentrated epidemics like that in the U.S. require HIV testing strategies that engage marginalized populations who are not connected to healthcare, in addition to healthcare‐based routine screening to implement the CDC recommendation to be tested once in a lifetime for HIV [6, 7]. One potentially cost‐effective approach may be contact tracing (aka. partner services) [8]. Contact tracing is a risk‐network based approach to infectious disease elimination that has been utilized for decades. The process involves asking newly HIV diagnosed clients to identify their sex or drug contacts so that they can be informed they have been exposed, and receive testing and care. However, traditional contact tracing has produced a relatively low yield of identifying sexual contacts of newly HIV diagnosed clients. In a review of 51 public health jurisdictions, only 0.96 contacts were identified per index, with 61% being notified of potential exposure [9].

Contact tracing network approaches that include the recruitment of social contacts in addition to risk contacts, known as a Social Network Strategy (SNS) have been more effective, and have been recently promoted by the CDC [10]. This strategy identifies HIV‐positive individuals and individuals at‐risk for acquiring HIV and asks them to recruit their social network for testing in exchange for an incentive. Pilot data found that 6% of individuals tested through SNS were newly identified HIV infections, which is five times the prevalence found via publicly funded counselling, testing and referral sites [11]. However, data on the costs of broadly implementing SNS strategies are limited.

This analysis seeks to compare the effectiveness and cost of three HIV testing strategies that occurred simultaneously at an academic medical centre (AMC) in Chicago (a high HIV burden area in the U.S.) in identifying new HIV infections. These three strategies comprised: (1) HIV screening in an AMC emergency department, inpatient hospital and clinics (routine hospital‐based testing); (2) recruitment for testing of the risk contacts of clients who were recently infected or diagnosed with HIV (modified partner services) and (3) recruitment of social contacts for HIV testing (RDS‐based SNS).

## 2 METHODS

A costing analysis of three HIV testing strategies was conducted from a healthcare payer perspective. We utilized an ingredients‐based approach, where each cost component is identified and assigned a cost value [12]. Three HIV testing strategies occurred within the same timeframe at the same institution, allowing an opportunity for comparison.

### 2.1 Data

#### 2.1.1 Routine hospital‐based HIV testing

Expanded Testing and Linkage to Care (X‐TLC) is a large‐scale, multi‐site HIV screening programme for populations disproportionately affected by HIV‐infection in the south and west sides of Chicago. We focus on the AMC site that is common to all the testing strategies. Testing occurred February 2011‐December 2016, and included screening in the emergency department, inpatient and outpatient settings. Consent for HIV testing was obtained through opt‐in oral consent (prompted by reminders in the electronic medical record (EMR) system if the patient was 18 to 64 years of age and did not have a prior HIV antibody test result), or if clinically indicated. HIV antibody testing could be ordered from the prompt. Test technologies included standard third‐generation blood‐based enzyme immunoassay and confirmatory Western blot or fourth‐generation HIV testing with Multispot confirmation. In 2016, confirmatory viral load testing was also added to fourth‐generation HIV testing. No incentives were provided for testing.

#### 2.1.2 Risk network strategy (modified partner services)

Modified partner services testing data come from the Transmission Reduction Intervention Project (TRIP), a longitudinal network intervention designed to identify recently HIV‐infected persons (rather than undiagnosed infected individuals) using a combination of testing history and viral load [13, 14, 15]. Contact tracing of sex and drug‐use partners was used for recruitment. Index cases included those recently infected with HIV (previous nine months). In addition to two sets of matched controls: those who are recently diagnosed with HIV (previous nine months), but not recently infected and those who were HIV negative.

**Figure 1:**
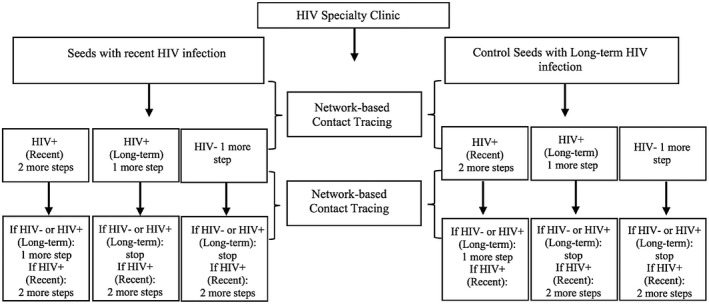
Arms and recruitment flow of the risk network strategy.

Index cases were recruited primarily from a HIV specialty clinic that was housed at the AMC; index controls were recently diagnosed, but had no documented evidence of seroconversion in the last nine months. Network and venue members of those recently infected and recently diagnosed were recruited using a two‐step algorithm designed to recruit people infected in the previous six months as previously described (Figure 1) [13]. Costs associated with viral load testing used to identify recently infected seeds were excluded. All respondents were ≥18 years old. Participants were given $50 for the baseline interview and $20 for each enrolled risk network member. Data in the analysis were collected September 2013‐February 2016. Blood samples were tested by AxSYM HIV‐1/2 gO (Abbott) and confirmed by Western Blot (MP Diagnostics) [13].

#### 2.1.3 Respondent driven sampling (RDS)‐based social network strategy

Social network testing data come from uConnect, a longitudinal study of young Black men who have sex with men (YMSM) ages 16 to 29 who reside in Chicago [16, 17, 18, 19]. Data were from the baseline visit, collected June 2013‐July 2014. RDS was used for recruitment. Index cases were selected from a distribution of social spaces that YMSM occupy (both physical and virtual spaces) and identified during focus groups. Respondents were required to: self‐identify as African‐American/Black, be born male, be 16 to 29 years of age, report oral or anal sex with a male within the past 24 months, and have a primary residence in South Chicago. Respondents were given up to six vouchers to recruit social network members who met the same criteria, $60 for the baseline interview and $20 for each enrolled social network member. HIV infection was determined by three assays applied to samples eluted from dry blood spots: ARCHITECT HIV Ag/Ab Combo; Multispot HIV‐1/HIV‐2 Bio‐Rad and Realtime HIV‐1 RNA, Abbot. Costs associated with viral load testing were excluded.

The Institutional Review Board at the University of Chicago and the National Opinion Research Center at the University of Chicago approved all procedures.

### 2.2 Analysis

#### 2.2.1 Measurement of costs

All costs were converted to 2016 U.S. dollars. Inputs were grouped into five categories; (i) personnel, (ii) HIV testing, (iii) training, (iv) materials and (v) overhead. Personnel for routine hospital‐based testing included direct service and support staff (caseworkers and/or clinical social workers, site administration and provider champions). Personnel for the modified partner services and social network strategies included direct service and support staff (site and database administrators and a project director). Each HIV testing and network elicitation session took approximately 60 to 120 minutes per participant for the modified partner services and social network strategies, accounting for 5% and 16% of the total personnel costs respectively. The remaining direct service and support staff time was spent on administrative and documentation tasks, including following up with participants about recruiting their network. Personnel costs were estimated using the U.S. Office of Personnel Management’s hourly rate of pay divisor [20]. Fringe benefits were included at 23.2% [21]. Training costs included training on the study procedures and an implementation phase where focus groups were held and seeds were selected. The cost of HIV testing was standardized across studies: $10 per rapid point‐of‐care test and $8.83 per blood test. These rates were established by Formedica, the laboratory accounting system at the University. We assumed a payer mix (observed at the AMC) of 25% Medicaid, 72% private and 3% uninsured patients across all programmes and applied the cost per test to the 3% uninsured.

Training costs were considered capital costs, depreciating as the duration of the programme increases. Materials costs for both the modified partner services and the social network strategy included standard costs (printing, phone, social media messaging charges), as well as incentives for the visit and referral of others. Overhead costs were included at 20% across all strategies [22]. A list of costs and assumptions can be found in Table 1. The main outcome of the analysis is cost per new HIV diagnosis. Secondary outcomes include the cost per HIV test and per HIV‐positive test result. The cost per HIV‐positive result was included to provide information on each strategy as it was implemented. While re‐engagement was not an objective of the study strategies, evidence suggests that repeat contact with clients may encourage engagement in care [23, 24]. An estimate of the cost of each HIV‐positive test may be beneficial for this effort elsewhere.

**Table 1:** Input variables and assumptions

Variable	Assumption
Labour	Occupational salaries were standardized across sites using data from the Bureau of Labor Statistics^14^ Fringe benefits were included at 23.2% [21]
Materials	Incentives were $50 for the study visit for modified partner services, $60 for the study visit in the RDS‐based social network strategy and $20 for network referrals in both programmes
Tests	Testing costs were standardized across sites at $10 per rapid point‐of‐care test and $8.83 per blood test, assuming 3% uninsured

### 2.3 Sensitivity analysis

Given differences in the patient populations between the strategies, we conducted a sensitivity analysis restricting the population to the ages and population groups seen in the most restrictive strategies. In essence, we limited all the tested populations to young, Black MSM and transgender women (see Table 3).

We also conducted a sensitivity analysis to approximate the implementation of these strategies in lower HIV prevalence populations. To do this, we used an HIV prevalence estimate of 23.4% for the social network strategy (the prevalence among MSM in the U.S. in 2016) [25] and a new diagnosis rate of 2.18 per 100 MSM; an HIV prevalence estimate of 0.037% for the routine hospital‐based HIV testing population (the prevalence among the general population in the U.S. in 2016) [26], and a new diagnosis rate of 0.0143 per 100 persons, and then decreased the HIV prevalence and new diagnosis rate among the modified partner strategy proportional to our observed difference in prevalence between the social network strategy and the modified partner strategy, assuming all three studies would be performed simultaneously at the same location. We assumed that the number of overall tests and the budget remained the same.

## 3 RESULTS

Demographics can be found in Table 2. Patients tested in the routine hospital‐based testing were mostly Black/African‐American (65%), female (63%) and >30 (median age 33, IQR 25 to 50). The routine hospital‐based testing programme completed 57,308 HIV tests among 43,249 people over the study period. This resulted in 360 (0.6%) HIV‐positive tests and 165 new HIV diagnoses (0.28%). Of the newly diagnosed HIV‐positive tests, 64 were MSM, 53 were Black/African‐American MSM and 36 were Black/African‐American MSM between the ages of 18 to 29.

**Table 2:** Demographics by HIV screening strategy

	Routine hospital‐based testing (n = 57,308)	Modified partner services (n = 146)	RDS‐based social network strategy (n = 508)
Age (median, IQR)	33 (25, 50)	26 (23, 31)	23 (20, 25)
<20	5248 (9%)	16 (11%)	77 (15%)
20 to 29	17,895 (31%)	84 (59%)	431 (85%)
30 to 39	12,961 (21%)	18 (13%)	0
40+	21,856 (38%)	25 (17%)	0
Missing	18 (0.03%)		
Race/ethnicity			
Black/African American	37,404 (65%)	129 (88%)	508 (100%)
White	12,961 (23%)	4 (3%)	8 (2%)^a^
Other	3525 (6%)	11 (8%)	22 (8%)^a^
Missing	3418 (6%)	**2 (1%)**	
Gender			
Male	20,915 (37%)	125 (86%)	471 (93%)
Female	36,388 (63%)	15 (10%)	0 (0%)
Transgender	5 (0.01%)	5 (3%)	36 (7%)
Missing		**1 (<1%)**	
Risk population			
Men who have sex with men	106 (0.2%)	121 (83%)	508 (100%)
People who inject drugs	23 (0.04%)	3 (2%)	4 (<1%)
Heterosexuals with multiple sexual partners	–	22 (15%)	0 (0%)

a Identified as both Black/African American and White/other race/ethnicity.

Modified partner services recruited 218 participants, 33 (15 %) of whom were HIV‐negative controls and excluded. Of the remaining participants, the majority identified as Black/African‐American (88%), male (86%) and <30 (median age 26, IQR 23 to 31). A total of 146 were tested for HIV over the time period (39refusedorwereunabletobetested). This resulted in 79 (54%) HIV‐positive tests and eight new HIV diagnoses (5%) overall.

All the RDS‐based social network testing participants identified as Black/African‐American (as a result of the inclusion criteria), were primarily male (93%), and <25 (median age 23, IQR 20 to 25). The social network strategy recruited 618 participants, and completed 508 HIV tests over the study period (110refusedorwereunabletobetested). This resulted in 210 (41%) HIV‐positive tests and 37 new HIV diagnoses (7.2%).

Overall cost breakdowns for each programme can be seen in Table 3. The overall cost for the routine hospital‐based testing programme was $2,767,481, with labour accounting for 74% of total costs. The overall cost for the modified partner services programme was $491,347, with labour accounting for 78% of total costs. The overall cost for the RDS‐based social network‐testing programme was $580,260 with labour (54%) and training (18%) accounting for most total costs. Consequently, the estimated cost per new HIV diagnosis was $16,773 for routine hospital‐based testing, $61,418 for modified partner services and $15,683 for RDS‐based social network testing. Estimated costs per HIV‐positive test were $7687 for routine hospital‐based testing, $6219 for modified partner services and $2763 for RDS‐based social network testing. Estimated costs per HIV test were $48 for routine hospital‐based testing, $2857 for modified partner services and $1142 for RDS‐based social network testing.

**Table 3:** Total programme costs, HIV testing results and cost analysis results

	Routine hospital‐based testing	Modified partner services (MPS)	RDS‐based social network strategy (SNS)	Sensitivity analysis		
				Routine hospital‐based Testing	MPS	SNS
HIV testing						
# Tested	57,308	146	508	3167	79	490
# HIV positive	360	79	210	81 (49 are MSM)	48	208
# Newly diagnosed	165	8	37	52 (36 are MSM)	8	37

	n (%)^a^	n (%)	n (%)	n (%)	n (%)	n (%)
Costs						
HIV testing costs	$18,844 (1%)	$79 (<1%)	$227 (<1%)	$1006 (1%)	$43 (<1%)	$219 (<1%)
Labour costs	$2,048,118 (74%)	$385,560 (78%)	$314,196 (54%)	$109,346 (74%)	$208,625 (78%)	$303,063 (54%)
Training costs	$222,492 (8%)	$8053 (2%)	$103,000 (18%)	$11,879 (8%)	$4357 (2%)	$99,351 (18%)
Materials costs	$16,780 (1%)	$15,765 (3%)	$66,127 (11%)	$896 (1%)	$8530 (3%)	$63,784 (11%)
Total programme cost^b^	$2,767,481	$491,347	$580,260	$147,752	$265,866	$559,699
Cost per outcome						
Cost per test	$48	$2857	$1142	$64	$3365	$1142
Cost per HIV‐positive test	$7687	$6219	$2763	$1824	$5539	$2691
Cost per new diagnosis	$16,773	$61,418	$15,683	$2841	$33,233	$15,127

a % of total costs for each programme

b Total costs equal sum of components plus 20% overhead.

### 3.1 Sensitivity analysis

Reducing the total costs to reflect the proportion of young, Black, MSM/Transgender participants resulted in an estimated cost per HIV‐positive test of $1824, and an estimated cost per new HIV diagnosis of $2841 in the routine hospital‐based programme, an estimated cost per HIV‐positive test of $5539, and an estimated cost per new HIV diagnosis of $33,233 in the modified partner services programme, and an estimated cost per HIV‐positive test of $2691, and an estimated cost per new HIV diagnosis of $15,127 in the social network strategy.

The sensitivity results mirroring the general population in the U.S. resulted in an estimated cost per HIV‐positive test and new HIV diagnosis of $131,784 and $337,909 in the routine hospital‐based strategy, $10,992, and $205,585 in the modified partner services strategy and $4884 and $52,417 in the social network strategy.

## 4 DISCUSSION

Our analyses showed that each strategy had variable efficacy and associated costs in identifying new HIV diagnoses among different groups at‐risk for HIV infection. The RDS‐based social network strategy yielded the highest proportion of new diagnoses, followed closely by the modified partner services programme. While the routine hospital‐based testing programme yielded the smallest proportion of new infections, it identified the highest number, with patients from the most diverse populations at‐risk for HIV (i.e. 44 women, 74 heterosexuals, four people who inject drugs (PWID) and 58 persons >40 years old). Both the RDS‐based social network strategy and the routine hospital‐based testing programme were comparable in the cost per new diagnosis identified in the populations they were designed to serve, indicating that either may be beneficial depending on the stage of the epidemic in a particular region.

Given the history of Black MSM/transgender women and their HIV risk and positivity in Chicago, we conducted a sensitivity analysis which restricted the study populations to Black men and transgender women between 18 to 29 years of age in order to examine this particularly high‐risk group.

The sensitivity analysis limiting all populations to young, Black MSM doubled the efficiency of the modified partner services programme, and increased the efficiency of the routine hospital‐based testing by tenfold. Alternatively, establishing an RDS‐based social network MSM programme that allows recruitment of all MSM might double the costs of case finding in this setting. While MSM < 30 accounted for the largest proportion of new HIV diagnoses in Chicago, it is also important to note that individuals 30 to 39 accounted for 27% of newly diagnosed HIV cases in 2017, and the highest proportion (31.2 %) of late diagnoses [27]. The routine hospital‐based testing was more likely to capture these groups.

While effective, limiting interventions to this specific sub‐population does not capture the Latinx population which accounted for the second highest percentage of HIV diagnoses and late diagnoses among all race/ethnicities in Chicago in 2017 (21% and 24.5% respectively) [27]. Heterosexuals are also excluded, who accounted for the second highest percentage of HIV diagnoses and late diagnoses among all transmission groups in Chicago in 2017 (19% and 26% respectively) [27].

In contrast, the sensitivity analysis using HIV prevalence and new diagnosis estimates from the general MSM and general population in the U.S. show that the cost per new HIV diagnosis is significantly higher across all programmes. This is to be expected as the prevalence and new diagnosis rates in these general populations are much lower than those seen in Chicago. There was also more variation in the cost, with the social network strategy having a much lower cost per new HIV diagnosis than the routine hospital‐based and modified partner services programmes.

Our results were similar to others in the U.S. comparing HIV testing strategies [28]. Results from Rhode Island reviled that partner services yielded a higher proportion of new HIV diagnoses compared to testing at community based and clinical settings, and that it was cost‐effective [28]. The cost per new diagnosis reported was also within a similar range as ours [28], and elsewhere ($2135 to $11,241 2017 dollars) [29].

Our results differ, however, from a previous study using similar methods in Odessa, Ukraine among PWID [30]. The previous study compared TRIP (modified partner services) implemented in Odessa to RDS to recruit PWID and Outreach Testing of PWID at community sites. Modified partner services yielded a higher proportion of undiagnosed positives (14.6%) than the RDS strategy (5.0%) or Outreach Testing (2.4%), and that the cost per undiagnosed HIV‐positive was less among modified partner services than the other two strategies [30]. These differences may be a result of the RDS strategy being risk based (participants were required to have injected drugs in the previous 30 days) versus social in this study. Differences in local contextual factors also likely play a role in these varied findings.

### 4.1 Study limitations

This study is a natural experiment where we were able to observe three different HIV testing programmes during roughly the same time period in the same AMC. As such, a rigorous comparison of the strategies was not possible, as the structure and target populations were different across strategies. We were also unable to detail labour time spent on various activities in each strategy, which may limit our ability to determine generalizability to localities with different labour cost structures.

It is possible that the yields of the different strategies could be confounded with secular changes in HIV patterns. We expect these biases to be minimal due to the overlapping time period of these three programmes, and the low PrEP uptake at the time they were conducted (<10% among young MSM in Chicago, <1% among heterosexuals nationwide in 2016) [31, 32]. In addition, the sensitivity analysis restricting the populations assumes that the programmes have low fixed costs. If fixed costs are high, this analysis understates average costs. It should also be noted that the network strategies tested all participants regardless of their self‐reported HIV status, which increased costs overall. Finally, incentivizing participation in testing and referral of peers in the network strategies may not be widely implementable in non‐research settings. However, we enumerated costs so others could evaluate site‐specific costs associated with implementation.

While our analysis strived to provide cost data for various screening approaches that may impact budget considerations by using a payer perspective, there are alternative perspectives for assessing cost‐effectiveness, like the societal perspective (when all costs and effects are incorporated no matter who pays the costs or receives the effects). The impact on long‐term life expectancy and quality of life is not explicitly valued in this analysis. Future analyses will work to integrate these perspectives and compare additional strategies.

Our analysis also likely underestimates total benefits received from all the HIV screening programmes because it does not evaluate the effect of re‐engaging those lost to HIV care [33]. Poor retention is both detrimental to those HIV‐infected and to preventing future transmission. While re‐engagement was not an objective of the study strategies, evidence from the CDC’s Data to Care initiative suggest that additional contact with clients may significantly increase re‐linkage to care [23, 24, 34, 35].

Future studies should evaluate the cost‐effectiveness of HIV retention interventions, and consider the cost per diagnosis of recent infection. While we were unable to measure this outcome, it may affect the relative social value of the different strategies. Diagnosing someone with recent infection may be worth more from an economic perspective than diagnosing someone with a late stage‐infection, as treating someone early would maximize the potential prevention of HIV transmission.

## 5 CONCLUSIONS

This analysis suggests that different strategies may be appropriate depending upon the phase of the epidemic. For instance in high incidence areas with general epidemics and limited personnel and resources, it may be more realistic to implement routine hospital testing with automatic alerts. Conversely, in areas with concentrated epidemics, social network strategies and modified partner services may better engage hard‐to‐reach vulnerable communities. New diagnoses ranged from 5% to 7.5% in the network strategies, indicating that this population was not likely to be captured by routine outpatient settings. This finding is corroborated by several others which demonstrate the success of network‐based testing in engaging at‐risk individuals with no previous testing history [36, 37, 38]. Given the geographic heterogeneity of the HIV epidemiology and the varied clinical settings in which testing may be implemented, a simultaneous approach may be the most effective and efficient at identifying new HIV infections in the U.S.

## COMPETING INTERESTS

The authors have to conflicts of interest to report.

## AUTHOR’S CONTRIBUTIONS

BS, MTP, HAP, SRF, DP and JAS contributed to the design and implementation of the research, to the analysis of the results. BS wrote the manuscript with consultation from MCM, EEF and JS. All authors have read and approved the final manuscript.
